# Supplementary material for: Glutamate oxaloacetate transaminase 1 is dispensable in macrophage differentiation and anti-pathogen response
Source: Commun Biol. 2024 Jul 5;7:817. doi: 10.1038/s42003-024-06479-w (PMC11224350; doi:10.1038/s42003-024-06479-w)

Supplementary Information for

Glutamate oxaloacetate transaminase 1 is dispensable in macrophage differentiation and anti-pathogen response

Lishan Zhang[1], Zhengyi Wu[1], Xuanhui Qiu[1], Jia Zhang[1], Shih-Chin Cheng[1,*]

[1] State Key Laboratory of Cellular Stress Biology, School of Life Science, Faculty of Medicine and Life Sciences, Xiamen University, Xiamen 361102, China

* Correspondence: jamescheng@xmu.edu.cn

This file includes:

Supplementary table 1-2 and Supplementary Figure 1-13

Supplementary Table 1. The sequences of *Got2* siRNA

	**sequences**
si*Got2*_1	CGAAGCCTTCAAGAGAGAT
si*Got2*_2	GGATGGCTGCTGCCTTTCA
si*Got2*_3	GAAGATGAACCTGGGAGTT
NC	The control siRNA was provided by RiboBio.

Supplementary Table 2. Primer sequence for real-time PCR

	**Primer Forward**	**Primer Reverse**
β2m	TTCTGGTGCTTGTCTCACTGA	CAGTATGTTCGGCTTCCCATTC
Got1	GCTGTGCTTCTCGCCTAGTT	AAGACTGCACCCCTCCAAC
Got2	ATGGCTGCTGCCTTTCAC	GATCTGGAGGTCCCATTTCA
Il6	TAGTCCTTCCTACCCCAATTTCC	TTGGTCCTTAGCCACTCCTTC
Tnfa	CCCTCACACTCAGATCATCTTCT	GCTACGACGTGGGCTACAG
Il1b	TTCAGGCAGGCAGTATCACTC	GAAGGTCCACGGGAAAGACAC
Arg1	CCAGAAGAATGGAAGAGTCAGTGT	GCAGATATGCAGGGAGTCACC
Chil3	CAGGTCTGGCAATTCTTCTGAA	GTCTTGCTCATGTGTGTAAGTGA
Retnla	TCCCAGTGAATACTGATGAGA	CCACTCTGGATCTCCCAAGA

Supplementary Figure 1 The construction, breeding strategy and genotyping of *Got1*$^{f/f}$ and *Got1*$^{\Delta LysM}$ mice.

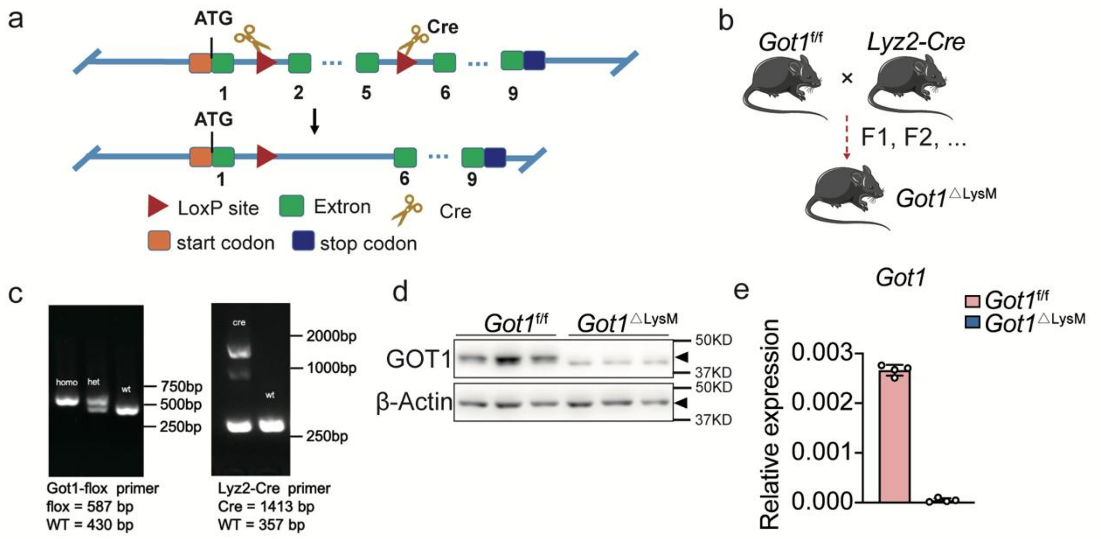

a, b, The construction and breeding strategy of *Got1*$^{f/f}$ and *Got1*$^{\Delta LysM}$ mice. c, Representative genotyping PCR gel electrophoresis images were shown. d, GOT1 protein level and e, *Got1* mRNA level in BMDMs derived from *Got1*$^{f/f}$ and *Got1*$^{\Delta LysM}$ mice.

Supplementary Figure 2 AOAA does not affect the expression of *Got1/2*, and PF-04859989 exhibits similar inhibition on pro-inflammatory macrophages as AOAA.

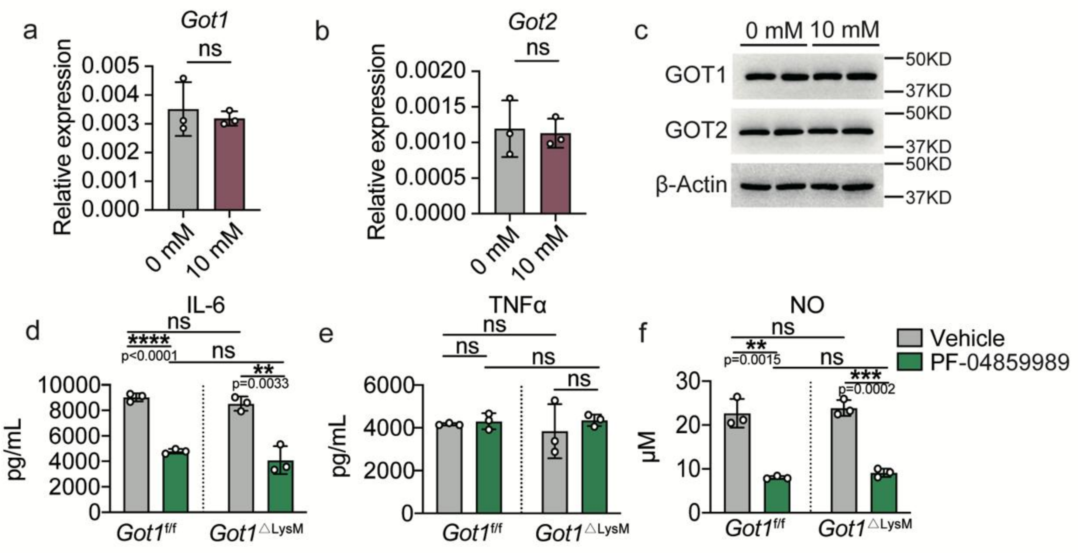

a, b, Real-time PCR showing no different between *Got1* and *Got2* mRNA levels of BMDMs treated with 10 mM AOAA for 24h. C57BL/6J mice, n=3 biologically independent experiments. c, Western blot showing no different between GOT1 and GOT2 protein levels of BMDMs treated with 10 mM AOAA for 24h. C57BL/6J mice, n=2 biologically independent animals. d-f, IL-6, TNFα and NO levels in BMDMs, derived from *Got1*$^{f/f}$ and *Got1*$^{\Delta LysM}$ mice, pretreated with 1 mM PF-04859989 for 24h, then treated with LPS (20 ng / mL) + IFNγ (100 ng / mL) for 24h. n=3 biologically independent experiments. ns, $p > 0.05$, not significant, **$p < 0.01$, ***$p < 0.001$, ****$p < 0.0001$, unpaired, two-tailed Student's t test. Data are representative of three independent experiments (mean ± SD)

Supplementary Figure 3 The production of pro-inflammatory cytokines in BMDMs is independent of *Got2*.

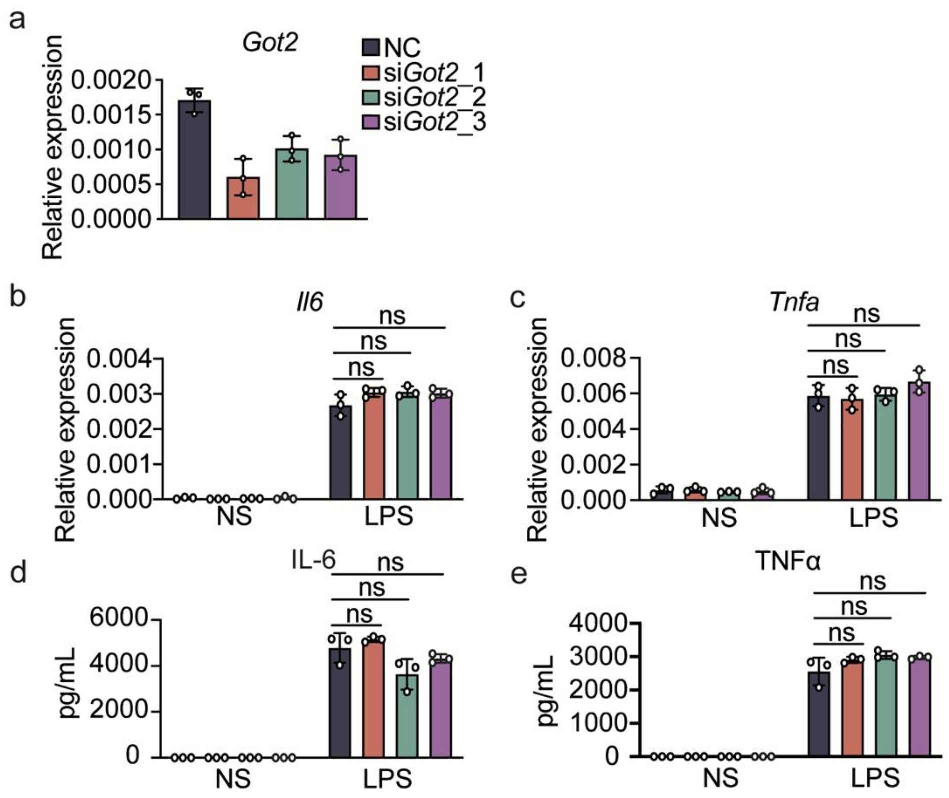

a. The knockdown efficiency of *Got2* in BMDMs transfection with *Got2* siRNA. d, e, *Il6* (b) and *Tnfa* (c) mRNA levels and f, g, IL-6 (d), TNFα (e) protein levels in the supernatant BMDMs derived from *Got1*$^{f/f}$ and *Got1*$^{\Delta LysM}$ mice. n=3 biologically independent experiments. ns, $p > 0.05$, not significant, unpaired, two-tailed Student's t test. Data are representative of three independent experiments (mean ± SD)

Supplementary Figure 4 Metabolic characterization of M1 macrophages treated with PF-04859989.

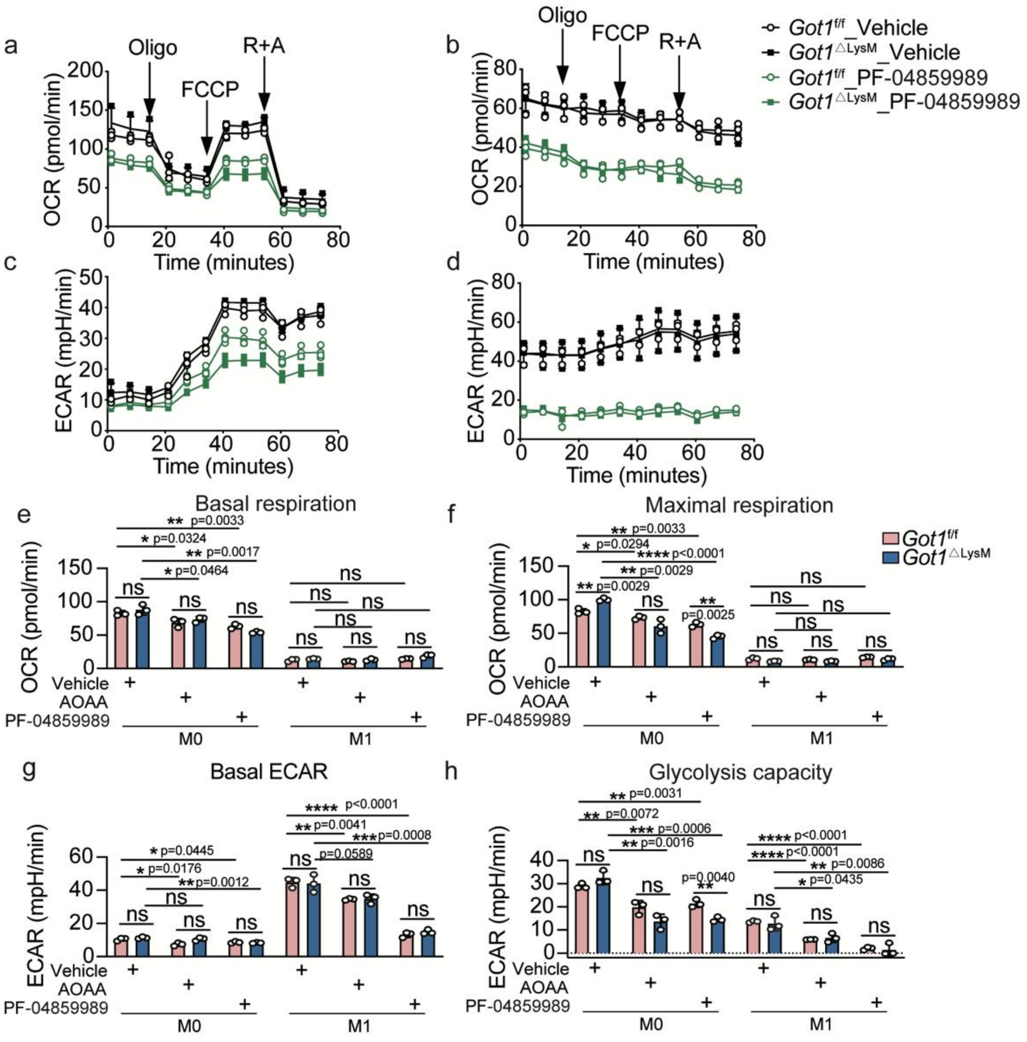

Seahorse analysis of OCR in M0 macrophages (a) and M1 macrophages (b), ECAR in M0 macrophages (c) and M1 macrophages (d), mitochondrial basal respiration capacity (e) and maximal respiration (f), basal ECAR values (g) and glycolysis capacity (h) were calculated. n=3 biologically independent samples. ns, $p > 0.05$, not significant, *$p < 0.05$, **$p < 0.01$, ***$p < 0.001$, ****$p < 0.0001$, unpaired, two-tailed Student’s t test. Data are representative of 3 samples per group (mean ± SD)

Supplementary Figure 5 *Got1* deficiency fails to influence the differentiation of pro-inflammatory macrophages while decreases ROS production in PMs.

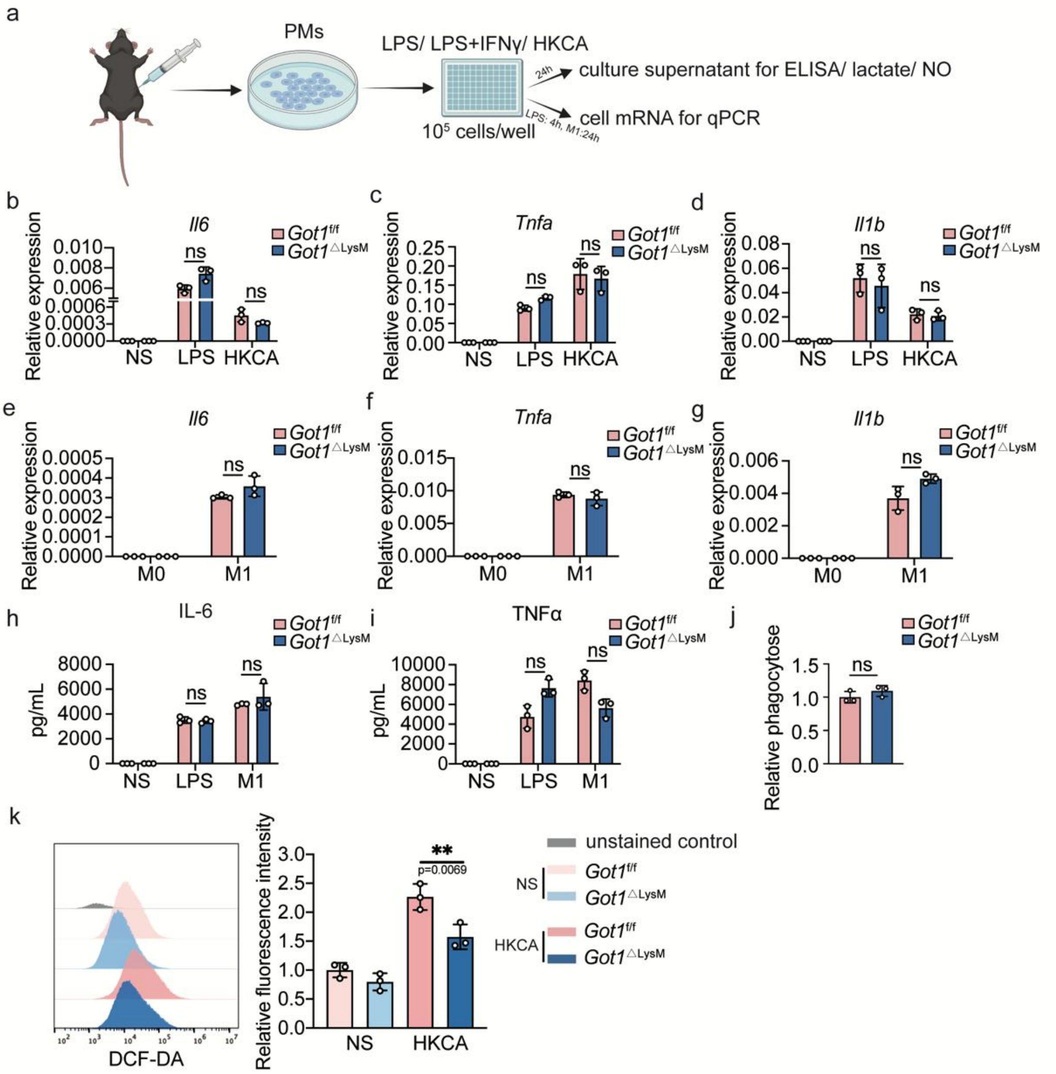

a, Schematic of study design. b-d, *Il6*, *Tnfa* and *Il1b* mRNA levels in PMs stimulated with LPS (100 ng / mL) or HKCA for 4h. *Got1*$^{f/f}$ mice, n=3 biologically independent experiments; *Got1*$^{\Delta LysM}$ mice, n=3 biologically independent experiments. e-g, *Il6*, *Tnfa* and *Il1b* mRNA levels in PMs stimulated with LPS (20 ng / mL) + IFNγ (100 ng / mL) for 24h. *Got1*$^{f/f}$ mice, n=3 biologically independent experiments; *Got1*$^{\Delta LysM}$ mice, n=3 biologically independent experiments. h, i, IL-6, TNFα protein levels in the supernatant determined by ELISA. j, Phagocytosis of PMs. FITC labeled-HKCA was utilized for phagocytosis assay with PMs. k, ROS production in PMs. Prior to ROS detection, PMs were pre-treated with HKCA (HKCA: BMDM=1:1) for 1h. *Got1*$^{f/f}$ mice, n=3 biologically independent experiments; *Got1*$^{\Delta LysM}$ mice, n=3 biologically independent experiments. ns, $p > 0.05$, not significant, **$p < 0.01$, unpaired, two-tailed Student's t test. Data are representative of three independent experiments (mean ± SD)

Supplementary Figure 6 The construction and breeding strategy of *Got1*$^{stop/+}$ and *Got1*$^{stop/+}$; *Lyz2-Cre* mice, while overexpression *Got1* failed to influence the ROS production.

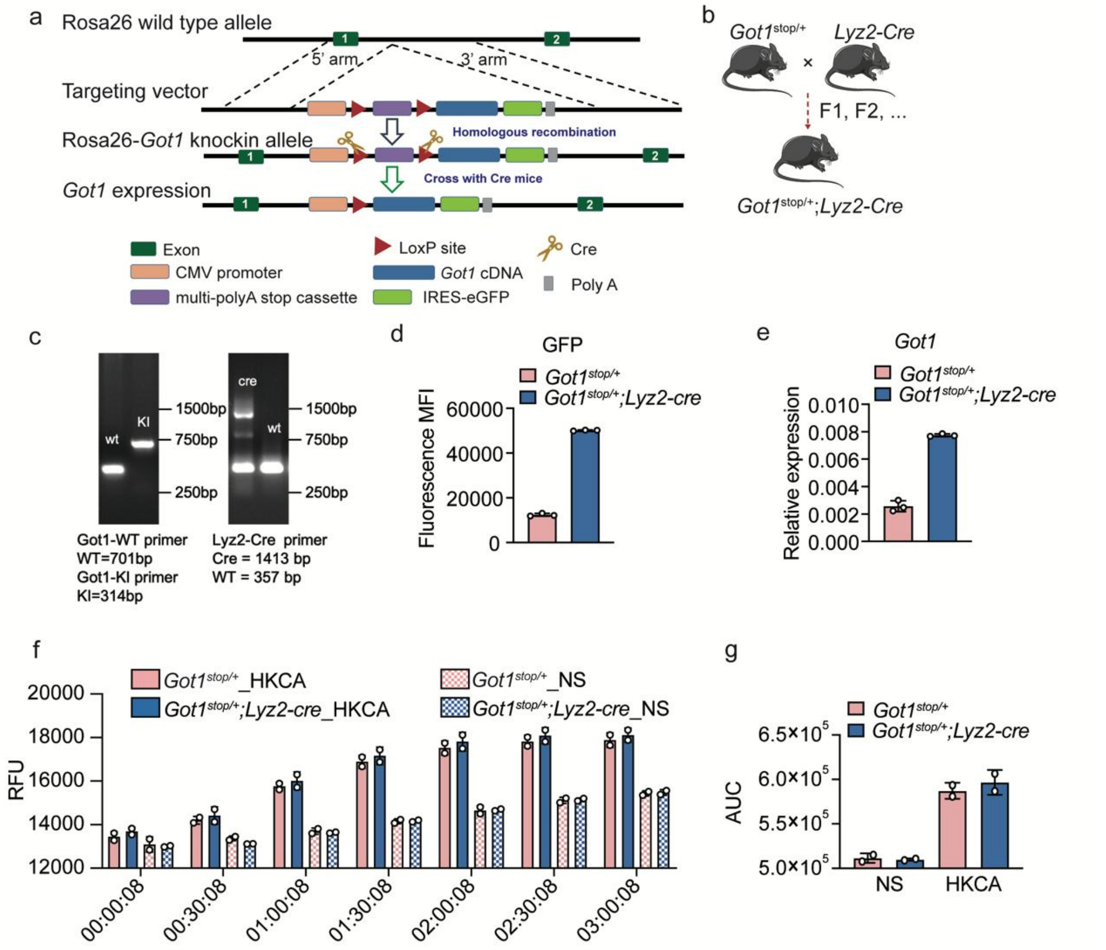

a, b, The construction and breeding strategy of *Got1*$^{stop/+}$ and *Got1*$^{stop/+}$; *Lyz2-Cre* mice. c, Genotyping PCR gel electrophoresis image was generated using genomic DNA. d, Fluorescence MFI of GFP in BMDMs. e, *Got1* mRNA levels in BMDMs. f, g, ROS production in BMDMs derived from *Got1*$^{stop/+}$ and *Got1*$^{stop/+}$; *Lyz2-Cre* mice.

Supplementary Figure 7 *Got1* depletion decreases ROS production in BMDMs, regardless of the addition of PF-04859989.

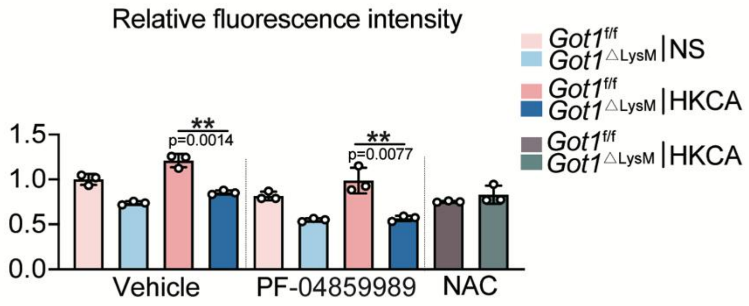

Prior to ROS detection, BMDMs were pre-treated with 1 mM PF-04859989 for 24h, then treated with HKCA (HKCA: BMDM=1:1) for 1h. $Got1^{f/f}$ mice, n=3 biologically independent experiments; $Got1^{\Delta LysM}$ mice, n=3 biologically independent experiments. **p < 0.01, unpaired, two-tailed Student's t test. Data are representative of three independent experiments (mean ± SD)

Supplementary Figure 8 The differentiation of M2 macrophages is independent of *Got1*.

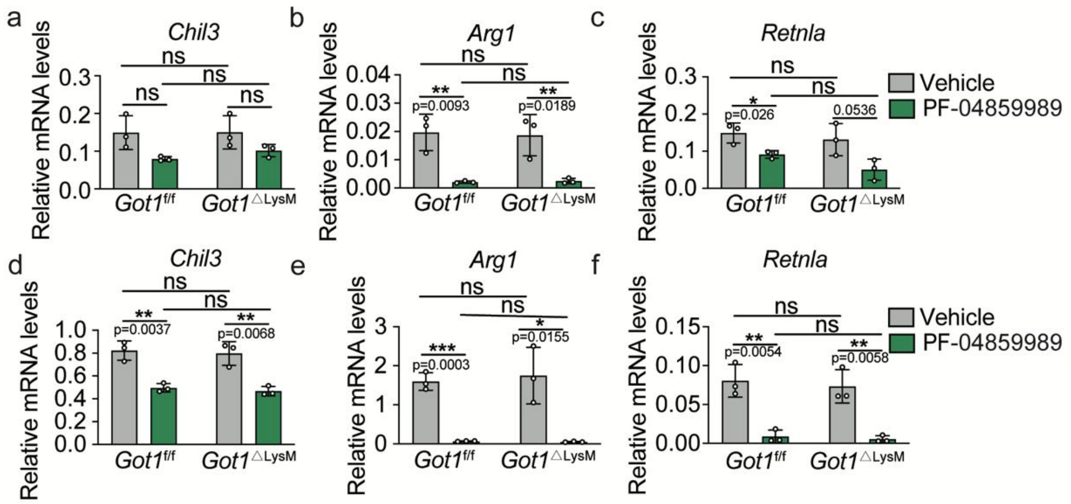

a-c *Chil3*, *Arg1*, and *Retnla* mRNA levels in BMDMs, derived from *Got1*$^{f/f}$ and *Got1*$^{\Delta LysM}$ mice, pretreated with 1 mM PF-04859989 for 24h, then treated with IL-4 (20 ng / mL) for 24h. n=3 biologically independent experiments. d-f, *Chil3*, *Arg1*, and *Retnla* mRNA levels in PMs, derived from *Got1*$^{f/f}$ and *Got1*$^{\Delta LysM}$ mice, pretreated with 1mM PF-04859989 for 24h, then treated with IL-4 (20 ng / mL) for 24h. n=3 biologically independent experiments. ns, $p > 0.05$, not significant, $^{*}p < 0.05$, $^{**}p < 0.01$, $^{***}p < 0.001$, unpaired, two-tailed Student’s t test. Data are representative of three independent experiments (mean ± SD)

Supplementary Figure 9 LPS induced immune tolerance in macrophages is independent of *Got1*.

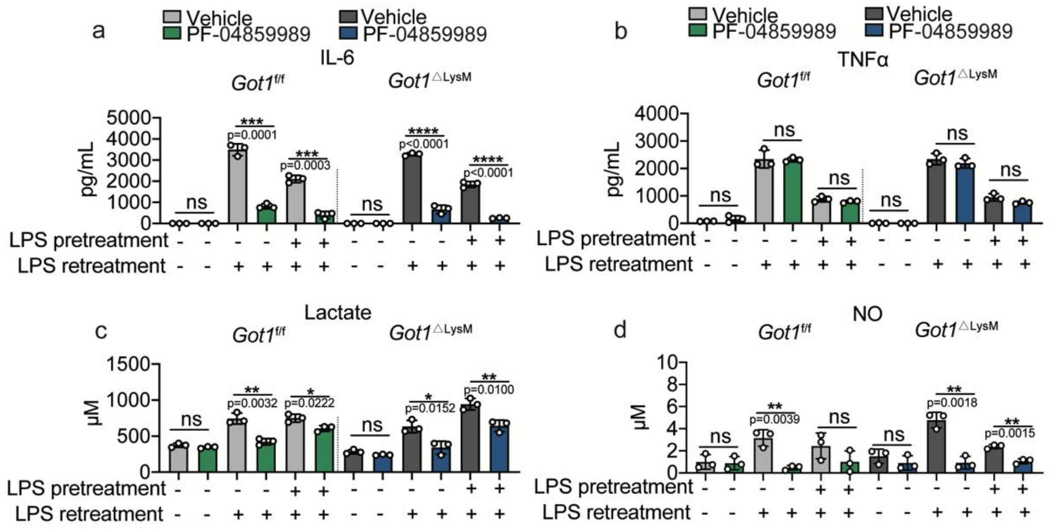

a, b, IL-6, TNFα protein levels in the supernatant of *Got1*$^{f/f}$ and *Got1*$^{\Delta LysM}$ mice derived BMDMs were determined by ELISA. Before LPS retreatment, BMDMs were pretreated with 1mM PF-04859989 for 24h, n=3 biologically independent experiments. c, Lactate production level in BMDMs stimulated with LPS (100 ng / mL) retreatment for 24h. d, NO production level in BMDMs stimulated with LPS (100 ng / mL) retreatment for 24h. n=3 biologically independent experiments. ns, $p > 0.05$, not significant, *$p < 0.05$, **$p < 0.01$, ***$p < 0.001$, ****$p < 0.0001$, unpaired, two-tailed Student's t test. Data are representative of three independent experiments (mean ± SD)

Supplementary Figure 10 *Got1* deficiency fails to influence *Got2* mRNA levels.

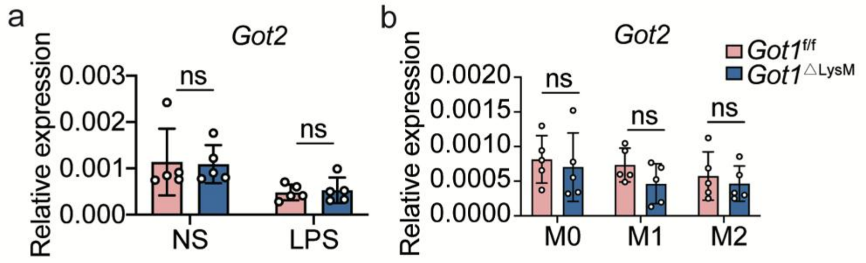

a, *Got2* mRNA level in BMDMs stimulated with LPS (100 ng / mL) for 4h. b, *Got2* mRNA level in BMDMs stimulated with LPS (20 ng / mL) + IFNγ (100 ng / mL) or IL-4 (20 ng / mL) for 24h. n=5 biologically independent experiments. ns, $p > 0.05$, not significant, unpaired, two-tailed Student's t test. Data are representative of three independent experiments (mean ± SD)

Supplementary Figure 11 The gating strategy of flow cytometry plots in Figure 4b, Supplementary Figure 5k and Supplementary Figure 7.

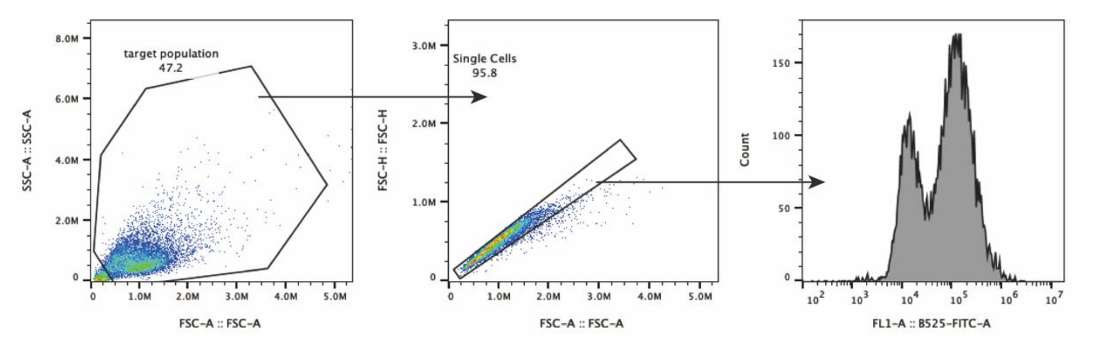

Supplementary Figure 12 The unedited images of western blotting in Supplementary Figure 1d and Supplementary Figure 2c.

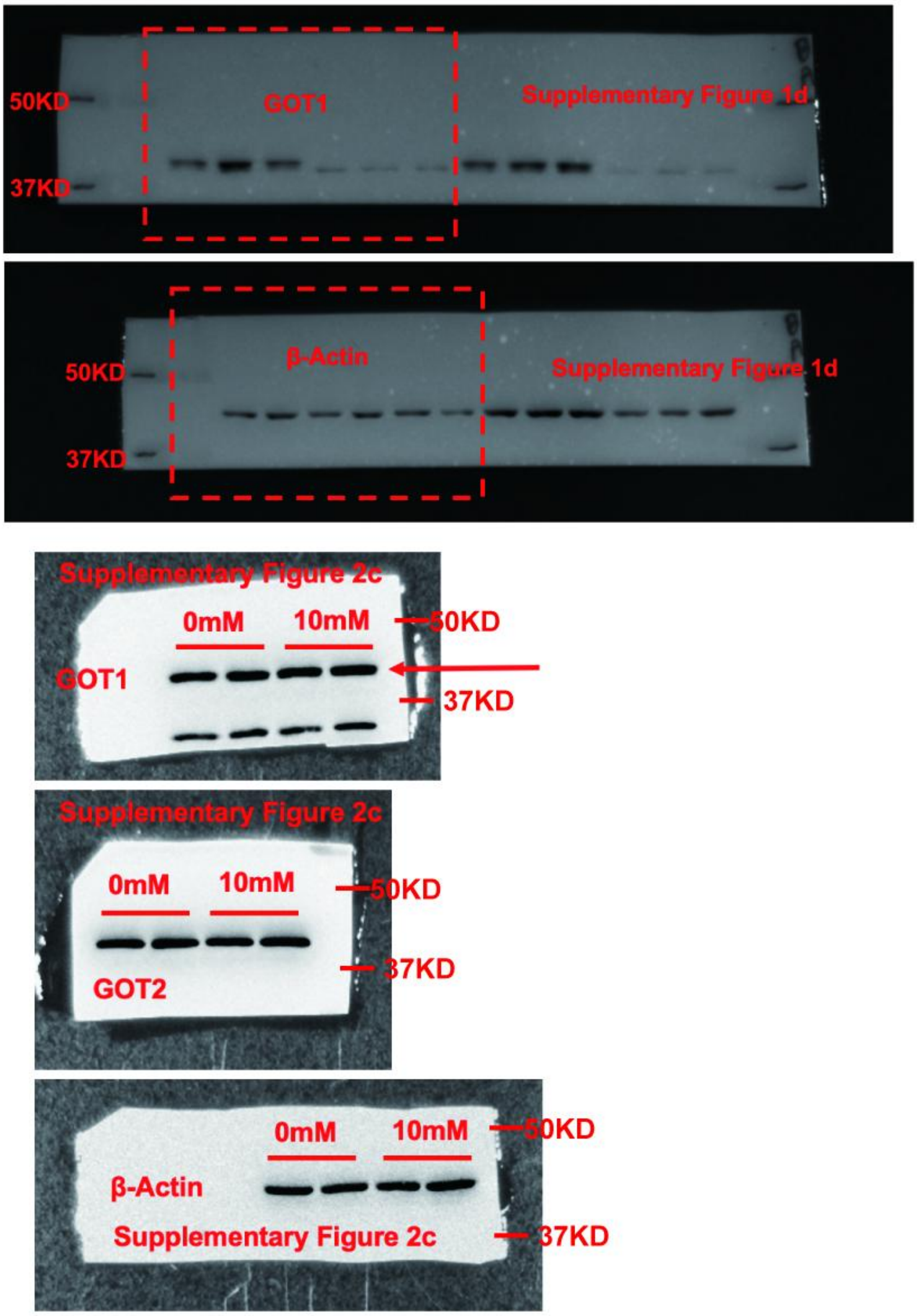

Supplementary Figure 13 The unedited images of DNA agarose gel in Supplementary Figure 1c and Supplementary Figure 6c.

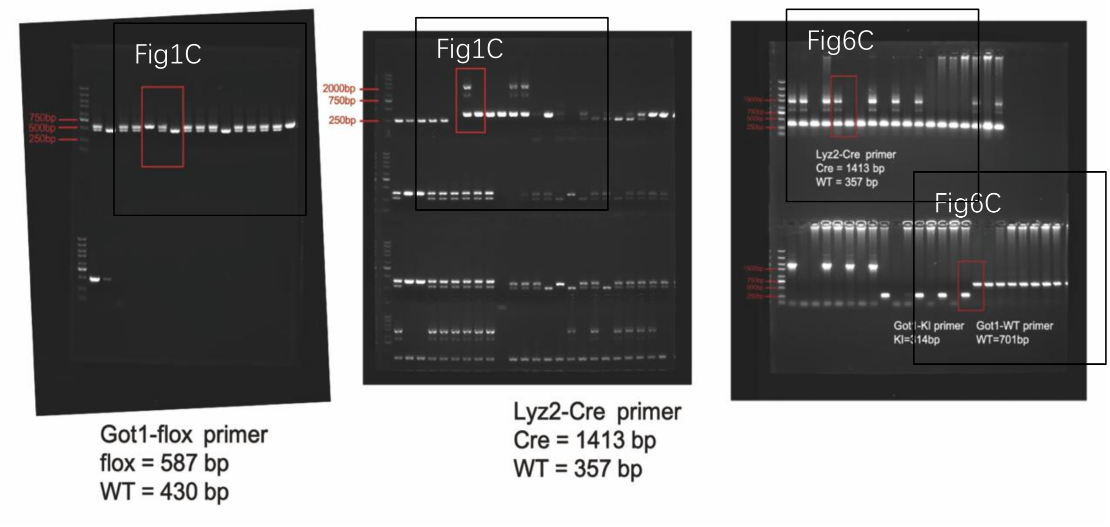

Supplement: Supplementary file 2 — Supplementary Information [file 42003_2024_6479_MOESM2_ESM.pdf]
